# Evaluation of Marginal and Internal Fit of Ceramic Laminate Veneers Fabricated with Five Intraoral Scanners and Indirect Digitization

**DOI:** 10.3390/ma16062181

**Published:** 2023-03-08

**Authors:** Ziad N. Al-Dwairi, Moataz Al-Sardi, Brian J. Goodacre, Charles J. Goodacre, Khaled Q. Al Hamad, Mutlu Özcan, Nadin Al-Haj Husain, Nadim Z. Baba

**Affiliations:** 1 Department of Prosthodontics, Faculty of Dentistry, Jordan University of Science and Technology (JUST), P.O. Box 3030, Irbid 22110, Jordan; 2 Division of General Dentistry, School of Dentistry, Loma Linda University, Loma Linda, CA 92350, USA; 3 Advanced Specialty Education Program in Implant Dentistry, School of Dentistry, Loma Linda University, Loma Linda, CA 92350, USA; 4 Division of Dental Biomaterials, Clinic for Reconstructive Dentistry, Center of Dental Medicine, University of Zurich, 8032 Zurich, Switzerland; 5 Department of Reconstructive Dentistry and Gerodontology, School of Dental Medicine, University of Bern, 3010 Bern, Switzerland; 6 Clinic of Masticatory Disorders, Center of Dental Medicine, University of Zurich, 8032 Zurich, Switzerland

**Keywords:** ceramic laminate veneer, dental materials, internal fit, intraoral scanner, marginal fit, prosthodontics

## Abstract

The long-term success of ceramic laminate veneers (CLVs) is influenced by the marginal and internal fit of the restorations. However, studies comparing the fit of CLVs using different intraoral scanners or the indirect digitization technique are lacking. The purpose of this study was therefore to assess the marginal and internal fit of CAD/CAM-milled CLVs using different intraoral scanners and the indirect digitalization technique. An ivorine typodont maxillary left-central incisor was prepared; the tooth and the neighboring teeth were scanned and used as a template to print ninety 3D partial models. Thereafter, ceramic laminate veneers (CLVs) (N = 90) were milled from IPS-Emax CAD blocks and divided into six equal groups (15 specimens each) according to the type of intraoral scanner (IOS), as follows: Omnicam IOS, SC3600 IOS, Trios 3 IOS, Emerald IOS, I500 IOS. Fifteen further CLVs were fabricated using the conventional indirect digitalization technique. After cementation on the resin dies and embedding in clear epoxy resin, specimens were sectioned inciso-gingivally and mesio-distally. At the incisal and cervical positions, the marginal discrepancy was measured and evaluated in addition to the internal gap at six locations using SEM (200×). Differences between gap measurements among the six groups were determined using ANOVA. Games–Howell multiple comparisons for homogenous variances and LSD multiple comparisons for non-homogenous variances were used with 95% confidence intervals. The significance level was set at 0.05. The lowest mean absolute marginal gap at the incisal margins (AMGI) was recorded for Omnicam group (203.28 ± 80.14) µm, while the highest mean absolute marginal gap at the cervical margins (AMGC) was recorded for Omnicam group (147.16 ± 59.78) µm. The mean AMGC was reported to be significantly different between the conventional technique (146.75 ± 38.43) µm and Trios 3 (91.86 ± (35.51) µm; *p* = 0.001) and between Emerald (112.37 ± (50.31) µm; *p* = 0.042) and I500 (86.95 ± (41.55) µm; *p* < 0.001). The mean MGI was found to be significantly different between the conventional technique (114.11 ± (43.45) µm and I500 group (186.99 ± (73.84) µm) only (*p* = 0.035). However, no significant differences were found in the mean MGI between all types of IOSs. The means of AMG and MG were significantly different at incisal or cervical areas between the conventional technique and IOSs and within the scanner groups (*p* > 0.05). Marginal gaps were higher in the incisal region compared to the cervical region with both the indirect digitization technique and the IOSs. Ceramic laminate veneers (CLVs) fabricated using IOSs produced overall internal and marginal fit adaptation results comparable to CLVs fabricated from the indirect digitalization method, and both techniques produced clinically acceptable results.

## 1. Introduction

Using computer-aided design and computer-aided manufacturing (CAD/CAM), the transformation of the clinical situation into a three-dimensional dataset in the production process of dental restorations can be achieved by direct or indirect digitalization [1]. Indirect extraoral digitalization starts with a conventional impression that is processed to a gypsum cast and then digitalized in the dental laboratory. In recent years, many new systems for direct intraoral digitalization have been introduced to dentistry with the aim of digitalizing the clinical workflow [2]. 

The success of any ceramic restoration is dependent on many factors, including its optical, mechanical, and bonding properties as well as its marginal and internal fit [3,4]. Marginal fit and microleakage have been used to assess the clinical success of cemented restorations for many years [5,6]. Success with bonded restorations such as ceramic laminate veneers depends on a number of factors, with marginal adaptation being one of the most important, and this is influenced by achieving contact between the tooth and veneer reconstruction [7,8,9]. 

As the composite resin cement is considered the weakest link in the ceramic veneer/resin cement/tooth complex, an intimate contact is highly recommended [10,11]. This weakness is caused by shrinkage of the luting composite during the polymerization process that could generate internal stresses which consequently from micro-cracks [12,13]. Under mechanical loading, these cracks propagate, resulting in restoration fracture [10]. Furthermore, the luting composite tends to water absorption when exposed to oral fluids, which results in dissolution of the resin matrix [14]. Furthermore, cement dissolution was enhanced by larger marginal gaps [15]. In addition, the difference in the coefficient of thermal contraction of the bonded surfaces may result in a marginal gap after exposure to hot and cold beverages and food in the oral cavity [16]. Therefore, it is recommended to have the highest adaptation possible of the veneer reconstruction to minimize the composite luting cement layer and possible oral environment exposure [10].

The values of fit reported by different studies have been widely variable [17]. Christensen et al. stated the clinically acceptable marginal gaps of gold inlays between 34 and 119 μm sub-gingivally and between 2 and 51 μm supragingivally [18]. Another study defined marginal gaps smaller than 120 μm [19], while other studies recommended fit values ranging from 7.5 μm up to 206.3 μm [20] as clinically acceptable. This variation could be explained by differences in the definition of fit, variations in fit measuring methods, and ceramic systems tested [20]. 

Several studies investigated the fit of restorations fabricated using intraoral scanners (IOSs) [17,21,22,23,24,25,26,27]. Zarauz et al. compared the fit of crowns fabricated using IOSs and the conventional impression replica technique with a stereomicroscopy [21]. The authors concluded that the fit values were significantly affected by the impression technique, with better fit of crowns fabricated using IOSs. However, the effect of cementation was not evaluated in that study. Mangano et al. compared twelve intraoral scanners by superimposing scanned models using best-fit algorithms on implants using different methods of assessment and concluded that different levels of trueness were found among IOSs [22]. Nagy et al. [23] compared seven types of IOSs and indirect digitalization methods on crown fabrication and concluded that errors existed in IOSs, with the indirect digitalization method being superior. In addition, Kwong et al. [25] compared the marginal gaps of CAD/CAM lithium disilicate crowns by using two different IOSs and found no significant differences in the mean marginal gap. 

As further studies comparing the fit of CLVs using different intraoral scanners or the indirect digitization technique are lacking, the purpose was to assess the marginal and internal fit of CAD/CAM-milled CLVs using different intraoral scanners and the indirect digitalization technique. To the best of the authors’ knowledge, no previous studies have compared IOSs and indirect digitalization for the fabrication of ceramic laminate veneers (CLVs) by assessing the marginal and internal fit of the veneers. Therefore, the purpose of this in vitro study was to assess the marginal and internal fit of CLVs using indirect digitalization and the direct digital technique by using five different IOS devices. The chosen methodology is evaluation by scanning electron microscopy, resulting in greater resolving power with greater field depth and higher magnification and sub-nanometer resolution compared to conventional digital microscopy. 

## 2. Materials and Methods

### 2.1. Specimen Preparation

**Figure 1:**
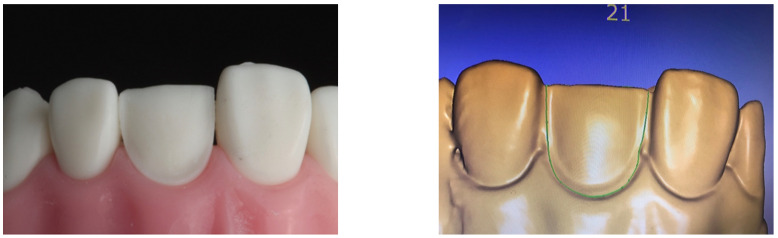
Veneer preparation on Ivorine typodont model and the corresponding digital scan.

An Ivorine typodont maxillary left-central incisor (Model number T860, Columbia Dentoform Teaching solutions, Long Island City, NY, USA) was used for the CLV tooth preparations. A conventional veneer preparation was completed using a Ceramic Veneer System (CVS) preparation bur set (CVS for porcelain veneers, Komet, Germany) (Figure 1).

Depth orientation grooves were prepared, followed by veneer preparation for a CLV restoration with a tapered diamond point and finishing stones. The reduction at the facial surface of the tooth was 0.4 mm in the cervical third with a chamfer finish line. The tooth preparation ended 1.0 mm occlusal to the cemento-enamel junction. At the middle and occlusal third, the reduction was 0.6 mm, with a butt joint incisal design to ensure the correct seating of the CLVs. The tooth preparation was performed interproximally without contact area removal. 

### 2.2. Specimen Fabrication

All experimental procedures were performed by one calibrated operator.

**Figure 2:**
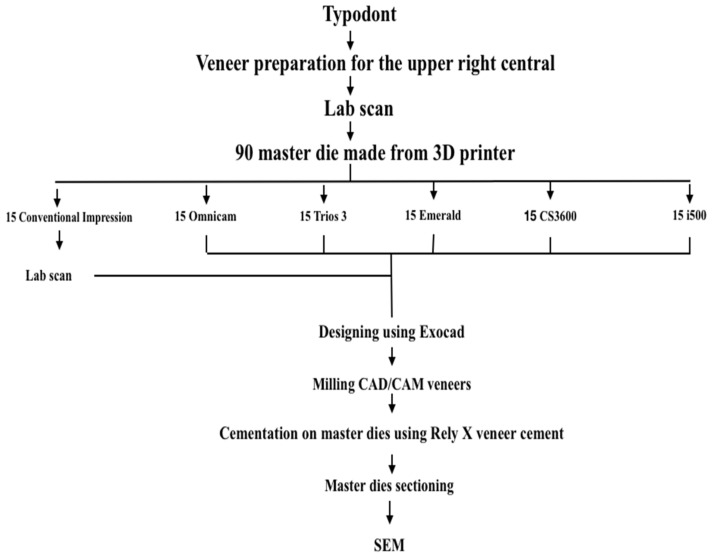
Schematic representation of sampling methodology.

The model, including the prepared tooth, was scanned with a laboratory scanner (Ceramill map 400, Amann Girrbach AG, Koblach, Austria). Ninety partial models, including the prepared and neighboring teeth, were printed by using a 3D print resin (NextDent for Ceramill 2.0, NextDent B.V, Conturionbaan, Soesterberg, The Netherlands) and a 3D printer machine with 50µm layer thickness (NextDent 5100, NextDent B.V). A conventional impression was made of 15 printed master dies using polyvinyl siloxane impression material (Elite HD plus, Zhermack, Badia Polesine, Italy) and poured using Type IV die stone (Jade Stone; Whip Mix Corp, Louisville, KY, USA). The 15 die stone casts were scanned using the laboratory scanner (Ceramill map 400 scanner, Amann Girrbach AG, Pforzheim, Germany), and a standard tessellation language (STL) file was obtained for each cast. Digital intraoral scans were made using five different IOSs (15 per group for a total of 75 scans): Cerec Omnicam/Dentsply Sirona scanner (Dentsply Sirona, New York, NY, USA), Trios 3/3 Shape scanner (Copenhagen, Denmark), CS3600/Carestream scanner (Atlanta, GA, USA), Emerald/Planmeca (Helsinki, Finland), I500/Medit (Merz Dental GmbH, Lütjenburg, Germany). In total, 90 STL files were obtained (15 from the indirect digitization technique with the laboratory scanner and 75 from the direct digitization techniques with the IOSs) (Figure 2). Sample size was chosen according to a previously published study [16].

**Figure 3:**
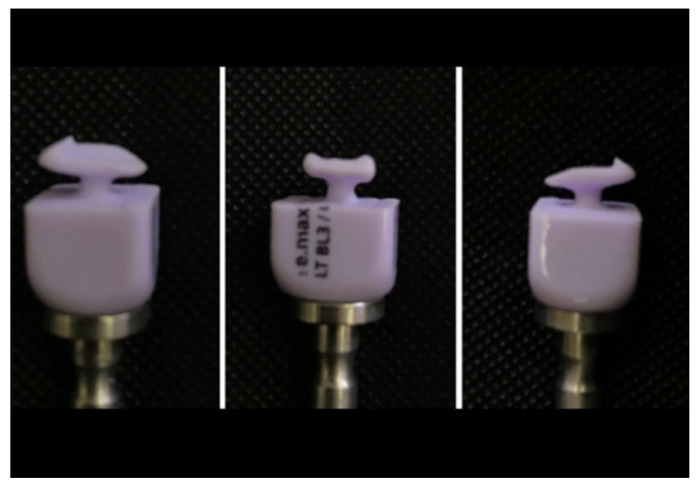
Milling the PLVs; IPS-Emax CAD blocks after milling.

Veneers were designed using a dental design software program (Exocad, Exocad GmbH, Darmstadt, Germany) and fabricated using lithium disilicate ceramic blocks (IPS-Emax CAD blocks, Ivoclar Vivadent, Schaan, Leichtenstein) with a five-axis milling machine (Ceramill^®^ Motion 2, Amann Girrbach AG, Germany). The lug was cut off, and any irregularities were removed and polished using a low-speed fine diamond bur for each veneer (Meister Point, Noritake Inc., Nagoya, Japan) (Figure 3). All specimens were sintered according to the manufacturer’s instructions.

The inner surface of each veneer was treated with 9% hydrofluoric acid solution for 20 s, washed with water for 10 s, dried with oil-free air for 10 s, and cleaned with a moist micro brush. Then, a silane coupling agent (Bisco Inc., Schaumburg, IL, USA) was applied for 1 min and allowed to dry. The resin dies were treated with 37% phosphoric acid (Bisco Inc., Schaumburg, IL, USA) for 20 s, washed with water for 10 s, dried for 10 s, treated with a universal adhesive (Scotchbond, 3M ESPE, Dental Products, MN, USA) for 20 s, and air dried for 5 s. Finally, an adequate amount of a light-polymerized resin cement (Rely X Veneer, 3M Espe AG, Espe Platz Seefeld, Germany) was applied to the inner surface of the veneer, and the veneer was placed on its corresponding die with a finger pressure, a process replicating the usual clinical technique. Short initial light curing with a wavelength of 420 nm to 480 nm and light intensity of 1000 mW/cm^2^–1200 mW/cm^2^ (Woodpecker LEDB curing light, Guilin Woodpecker medical instrument CO. LTD) was applied to the middle of the tooth surface to facilitate removal of excess cement. Final curing was performed later by applying the light source for 40 s from the facial and lingual surfaces.

### 2.3. Scanning and Morphology Analysis

For the scanning procedure, specimens were inserted in the middle of the prefabricated molds and fixated into blocks using clear epoxy resin (Ortho-Jet, Lang Dental Manufacturing Company, Wheeling, IL, USA). The blocks were mesio-distally and inciso-gingivally cut at the tooth surface center and perpendicular to the margins with a sectioning machine (IsoMet 1000 low-speed saw, Buehler, Germany). A scanning electron microscope (SEM) (Quanta 450 FEG, FEI) was used to examine sectioned specimens at a 200× magnification.

The cement layer thickness was measured at 8 points, including 2 marginal and 6 internal measurements. As for the marginal fit, marginal gap (MG) and absolute marginal gap (AMG) were obtained following Holmes et al. [28].

AMG was defined as the distance between the internal edge of the margin and the preparation finish line, while MG was set as perpendicular distance from the internal surface of the coping to the margin preparation and evaluated at the incisal and cervical margin [28]. The internal gap (IG) was defined according to Holmes et al. as the distance between the internal coping surface to the axial wall of the preparation [28]. Six internal measurement points were defined for each veneer at the following distances: 1.0 mm from the cervical margin, at the middle of the tooth inciso-gingivally (incisal quadrant), at 1.0 mm from the middle point toward the incisal, at the middle of the tooth inciso-gingivally (cervical quadrant), and at a distance of 1.0 mm from the middle point toward the cervical.

### 2.4. Statistical Analysis

At six different locations, the five parameters, namely (1) absolute marginal gap at the incisal edge (AMGI), (2) marginal gap at the incisal edge (MGI), (3) absolute marginal gap at the cervical (AMGC), (4) marginal gap at the cervical (MGC), and (5) internal gap (IG) were evaluated for each specimen. IBM SPSS version 25 statistical analysis software was used for data analysis; statistical differences among the groups were tested using one-way analysis of variance (ANOVA). Then, Games–Howell multiple comparisons with a 95% confidence interval were performed to compare the MGI in all groups with non-homogeneous variances. For MGC, AMGC, and IG, the Least Significant Difference (LSD) Post-hoc test was used with homogeneous variances, and *p*-values of ≤0.05 were considered significant. 

## 3. Results

**Figure 4:**
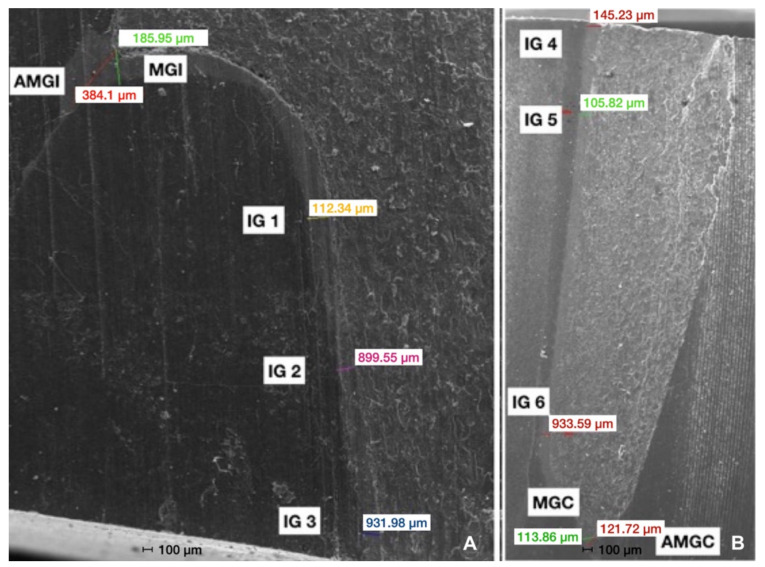
(**A**) Digital image of the incisal section. AMGI: Absolute Marginal Discrepancy at the Incisal edge. MGI: Marginal Gap at the Incisal edge. IG: Internal Gap; IG1 was measured at 1 mm distance from the incisal edge, IG2 was measured at 1 mm distance from the midline section toward the incisal, and IG3 was measured at the midline section. (**B**) Digital image of the cervical section. AMGC: Absolute Marginal Discrepancy at the Cervical edge. MGC: Marginal Gap at the Cervical margin. IG: Internal Gap; IG4 was measured at the cervical midline section, IG5 was measured at 1 mm distance from the midline section toward the cervical, and IG6 was measured at 1 mm distance from the cervical margin.

Figure 4 shows the incisal section of one specimen and all measuring points.

Mean values and standard deviations (SDs) of AMGI, AMGC, MGI, and MGC of all test groups are shown in Table 1. AMGI was affected by the intraoral scanner device used and showed the lowest mean values for the Omnicam group (203.28 ± 80.14 μm) and the highest mean for the Trios 3 group (289 ± 119 μm). AMGI was not affected significantly by type of IOS or indirect digitalization method (*p* = 0.149, F = 1.68). In contrast, the mean AMGC was significantly affected by type of IOS and indirect digitalization method (*p* < 0.001, F = 6.02). Similarly, the mean MGI was significantly affected by IOS type and indirect digitalization method (*p* = 0.002, F = 4.20), as were MGC (*p* = 0.029, F = 2.65) and IG (*p* < 0.001, F = 6.75). The LSD comparison test was used to detect differences between means of AMGC in different groups using both methods and is shown as superscript letters in Table 1. 

A comparison of the mean values of all groups (AMGI, MGI, AMGC, MGC, IG) between the indirect digitalization method and IOSs shows a significant difference in mean AMGC and MGI between indirect digitalization and IOSs, with indirect digitization producing smaller gaps (Table 2). 

The results showed that position had no significant effect on the AMG (*p* = 0.055, F = 3.74) (Table 3).

The results showed that position had a significant effect on the MG (*p* = 0.025). 

A comparison of the mean (AMGI, MGI, AMGC, MGC, IG) was observable with a significant difference between the groups AMGC and MGI.

## 4. Discussion

This study investigated the effect of different scanning techniques on the marginal and internal fit of veneer restorations cemented on resin dies. The results of the present investigation showed that when comparing IOSs with indirect digitalization, significant differences were reported in only the mean AMGC and MGI. However, when comparing the means between IOS and the indirect digitalization, all were significantly different except for the mean AMGI.

Several studies evaluated the fit of crowns using IOSs [21,24,25,26]. Some studies compared the trueness of multiple IOSs by using inspection and metrology software programs [22,23]. The results of the present study were consistent with a study by Mangano et al. [22]. They found significant differences between different IOSs and concluded that different levels of trueness were found among IOSs. Nagy et al. [23] compared seven types of IOSs and indirect digitalization on crown fabrication and concluded that errors existed in IOSs and reported superiority of the indirect digitalization method. Kwong et al. [24] compared the marginal gaps of two different IOSs and found no significant differences in the MMG when fabricating CAD/CAM lithium disilicate crowns. The mentioned studies assessed trueness of different IOSs and used inspection software programs for overlapping scans of crowns or implants, which was different from the methodology used in the present study.

Furthermore, when considering incisal versus cervical position, the results showed that the mean AMG was significantly affected in all groups using indirect digitalization and IOSs, whereas mean marginal gap (MG) was only significantly affected when using Omnicam, Trios 3, Emerald, and I500 IOSs. Both mean AMG and MG were higher at the incisal position compared to the cervical position, possibly due to the finishing and polishing of the cervical area. The results of this study were also consistent with several other studies that reported higher AMG at the incisal position compared with the cervical location [29,30]. Ranganathan et al. [31] tested different cervical and incisal marginal discrepancies using SEM. The higher values of the incisal gap in the present study could be related to the diameter of the milling tool that may be larger in diameter than some parts of the tooth preparation, such as the inner surface of the incisal edge, thereby causing a larger gap at the incisal margins [32]. Contrary to the results of the present study, Lin et al. [33] found the smallest mean vertical gap at the incisal location and the largest gap at the mesial. This could be related to a different research methodology used in their study, where the marginal discrepancy was evaluated with a replica technique and cross-sectional view using a digital microscope.

Tooth preparation in two planes and the convex nature of the tooth might explain the varying level of adaptation of the CLVs in different areas of the tooth. When the sharp, abrasive diamond instruments that are used in the milling become worn from heavy previous use, this will result in marginal chipping of ceramic veneer edges, resulting in higher gap measurements. Moreover, marginal discrepancy may arise from overgrinding by the bur due to its own diameter and chipping thin porcelain margins by the brittle nature of the material itself, as well as milling vibration. Since veneers demand a scrupulous geometric reduction, any shortcomings in these manufacturing steps affect the complete marginal integrity [32].

Different values for marginal fit have been reported throughout the literature. However, although 100 to 150 μm has been recommended by several authors as clinically acceptable with regard to longevity [18,19,20], one study reported that a 300 μm marginal discrepancy could be acceptable for ceramic restorations [33]. The mean marginal discrepancy varied between 125 and 402 μm at the incisal margin in one previous study [29]. Despite variable gap widths when different fabrication techniques and luting materials were evaluated, the mean values for the different groups observed in this study were as follows: 203–289 μm for AMGI, 87–147 μm for AMGC, 114–187 μm for MGI, 72–106 μm for MGC, and 71–104 μm for IG. Those values were close to values reported in previous studies, with significant differences between types of IOSs used for all tested points, except for AMGI.

Al-Dwairi et al. [27] examined the influence of fabrication technique and type of cement on the marginal and internal accuracy of porcelain laminate veneers in an in vitro study. The authors concluded that fabrication technique and type of cement significantly affected the marginal fit of the restorations. Furthermore, the marginal gap was found to be greater at the incisal aspect of the veneer than at the cervical position, as reported in the present study and previously mentioned studies. Photopolymer resin dies were used in this study as teeth analogues because they were able to adhere efficiently to resin luting cement. Natural teeth and metal or resin dies have been used in previous studies for assessment of fit [32,33]. However, natural teeth are widely variable because of their different dimensions, age, storage time, and medium after extraction. In contrast, resin dies have uniform dimensions and homogenous structure, which offers a reproducible medium for bonding [33,34]. 

CLVs were embedded in cubes of clear epoxy resin to avoid chipping during sectioning. The absolute marginal discrepancies and marginal gaps are not easy to evaluate by direct inspection or with scanning electron microscopy since the gap and even overhangs could be masked by the smooth surface of the luting agent. Therefore, sectioning of the specimens is preferred [29]. Evaluation of the internal gap in this study represented the cement film thickness and level of adaptation of the restoration to its corresponding die, whereas the AMG represented the marginal gap between the tooth and the prosthetic restoration [29,32].

The final accuracy of an IOS depends on several factors such as the operator, light conditions, excessive reflection due to metallic restoration, excessive saliva, or areas with poor access [35,36]. Obstruction of light may cause shadowing and loss of the areas that are not properly exposed such as steep surfaces, sharp edges, proximal areas, or subgingival margins [37]. Furthermore, a learning curve of the intraoral scanning procedure has been observed with an increase in scanning speed with more scanning experience [35]. In the present study, all models were captured by the same operator, environment, and light condition on a plastic model to eliminate any confounding factor.

Limitations of the present study included that the cementation of CLVs was performed under finger pressure, which is representative of procedures used clinically but does not guarantee complete uniform seating of the veneer. In future studies, it is suggested to use a standardized device to control the amount of pressure applied over each veneer during the cementation procedure. Furthermore, as this is an in vitro study, the factors that might affect the scanning procedure inside the patient’s mouth were not considered, nor was the accuracy of scanning of polymeric models using various devices and manufacturers. Blinding of the operator might lower the risk of bias, while further imaging methods could be applied to better understand the results. 

## 5. Conclusions

Ceramic laminate veneers fabricated using IOSs produced overall internal and marginal fit adaptation results comparable to the ones fabricated from the indirect digitalization method, and both techniques were effective, especially when the absolute marginal gap at the incisal margins was assessed. Furthermore, comparable results were found with both techniques for marginal gap at the incisal margins except for I500 scanner and Emerald scanners with marginal gap at the cervical margins and internal gap assessment. IOS systems showed more significant differences for absolute marginal gap at the cervical margins and when the position (incisal or cervical) of absolute marginal gap and marginal gap were considered.

## Figures and Tables

**Table 1:** Means (µm) and SDs for Absolute Marginal Gap at the Incisal Margins (AMGI), Marginal Gap at the Incisal Margins (MGI), Absolute Marginal Gap at the Cervical Margins (AMGC), and Average Internal Gap (IG) groups.

AMGI		MGI		AMGC		MGC		IG	
OM	203 ± 80	ID	114 ± 44 ^a^	I5	87 ± 42 ^a^	I5	72 ± 39 ^a^	OM	71 ± 19 ^a^
ID	228 ± 100	SC	128 ± 37 ^ab^	TR	92 ± 36 ^a^	EM	80 ± 22 ^ab^	SC	78 ± 17 ^ab^
Em	254 ± 123	OM	137 ± 55 ^ab^	EM	112 ± 50 ^ab^	TR	80 ± 34 ^ab^	TR	82 ± 13 ^ab^
I5	272 ± 88	EM	175 ± 96 ^ab^	SC	146 ± 39 ^bc^	ID	99 ± 24 ^bc^	ID	86 ± 15 ^bc^
SC	277 ± 64	I5	187 ± 74 ^b^	ID	147 ± 38 ^c^	OM	101 ± 32 ^bc^	I5	87 ± 16 ^bc^
TR	289 ± 119	TR	265 ± 206 ^b^	OM	147 ± 60 ^c^	SC	106 ± 40 ^c^	EM	104 ± 20 ^d^
p *	0.149		0.002 *		*p* < 0.001 *		0.029 *		*p* < 0.001 *

* ANOVA test; Values in the same column with at least one same superscript letter are not statistically significant using LSD test. Lower case letters indicate statistically similar groups in each column. (α = 0.05). OM: Omnica; ID: Indirect digitization; Em: Emerald; I5: I500 Medit; SC: Sc3600 Carestream; TR: Trios 3.

**Table 2:** Two-way ANOVA results of testing the significance of position and method on AMG.

Source	Type III Sum of Squares	df	Mean Square	F	Sig.
Corrected Model	804,024,192,816 ^a^	3	268,008,064,272	43.199	0.000
Intercept	3,519,418,862,453	1	3,519,418,862,453	567.276	0.000
Groups	6,886,437	1	6,886,437	0.001	0.973
Position	312,380,459,888	1	312,380,459,888	50.351	0.000
Groups/Position	23,172,179,553	1	23,172,179,553	3.735	**0.055**
Error	1,085,712,451,683	175	6,204,071,152		
Total	8,235,091,288,697	179			
Corrected Total	1,889,736,644,499	178			

AMG: Absolute Marginal Gap; ^a^. R Squared = 0.425 (Adjusted R Squared = 0.416); *p* value significant at 0.05.

**Table 3:** Two-way ANOVA results of testing the significance of position and method on MG.

Source	Type III Sum of Squares	df	Mean Square	F	Sig.
Corrected Model	328,112,780,394	3	109,370,926,798	16.282	0.000
Intercept	1,358,848,240,268	1	1,358,848,240,268	202.290	0.000
Groups	16,699,571,647	1	16,699,571,647	2.486	0.117
Position	66,213,915,509	1	66,213,915,509	9.857	0.002
Groups/Position	34,514,898,794	1	34,514,898,794	5.138	**0.025**
Error	1,162,096,008,687	173	6,717,317,969		
Total	4,430,775,443,978	177			
Corrected Total	1,490,208,789,081	176			

MG: Marginal Gap.

## Data Availability

Not applicable.
